# 3D Printing of Individualized Microfluidic Chips with DLP-Based Printer

**DOI:** 10.3390/ma16216984

**Published:** 2023-10-31

**Authors:** Jingjiang Qiu, Junfu Li, Zhongwei Guo, Yudong Zhang, Bangbang Nie, Guochen Qi, Xiang Zhang, Jiong Zhang, Ronghan Wei

**Affiliations:** 1 School of Mechanics and Safety Engineering, Zhengzhou University, Zhengzhou 450001, China; 2 Engineering Technology Research Center of Henan Province for MEMS Manufacturing and Applications, Zhengzhou University, Zhengzhou 450001, China; 3 Institute of Intelligent Sensing, Zhengzhou University, Zhengzhou 450001, China; 4 Department of Mechanical Engineering, College of Engineering, City University of Hong Kong, Kowloon Tong, Kowloon, Hong Kong, China; 5 Industrial Technology Research Institute, Zhengzhou University, Zhengzhou 450001, China

**Keywords:** 3D printing, microfluidic chips, DLP, direct printing, open channel design

## Abstract

Microfluidic chips have shown their potential for applications in fields such as chemistry and biology, and 3D printing is increasingly utilized as the fabrication method for microfluidic chips. To address key issues such as the long printing time for conventional 3D printing of a single chip and the demand for rapid response in individualized microfluidic chip customization, we have optimized the use of DLP (digital light processing) technology, which offers faster printing speeds due to its surface exposure method. In this study, we specifically focused on developing a fast-manufacturing process for directly printing microfluidic chips, addressing the high cost of traditional microfabrication processes and the lengthy production times associated with other 3D printing methods for microfluidic chips. Based on the designed three-dimensional chip model, we utilized a DLP-based printer to directly print two-dimensional and three-dimensional microfluidic chips with photosensitive resin. To overcome the challenge of clogging in printing microchannels, we proposed a printing method that combined an open-channel design with transparent adhesive tape sealing. This method enables the rapid printing of microfluidic chips with complex and intricate microstructures. This research provides a crucial foundation for the development of microfluidic chips in biomedical research.

## 1. Introduction

Microfluidics, which is also known as lab-on-a-chip or micro total analysis systems, refers to the technology platform that integrates the preparation, reaction, separation, and detection of samples in the fields of biology and chemistry into a single chip. This technology could realize the precise fluid manipulation and analysis at the micro scale [1]. Because of its advantages in low sample consumption, high precision in separation and detection, and low cost, microfluidic technology has been broadly applied in the fields of chemistry [2,3], biology [4,5], medicine [6,7], environmental science [8,9], etc. With the development of microfabrication technology, the fabrication methods of microfluidic chips have also developed rapidly.

While traditional microfabrication methods have made progress in decreasing the manufacturing cost and time of microfluidic chips, they still face several critical issues in the development of multifunctional integrated microfluidic chips. Firstly, in traditional methods such as soft lithography [10] and hot embossing [11], chip templates need to be prepared in advance through various means. The design and preparation of these templates determine the relatively long manufacturing cycle of microfluidic chips, resulting in high costs for customized chip production. Secondly, for the design of a three-dimensional microchannel, traditional methods can only achieve two-dimensional structures. Existing methods often rely on the complex assembly of two-dimensional structures in different planes, which is called stacking, to achieve the manufacturing of three-dimensional microfluidic chips [12].

In the past decade, 3D printing technology has experienced rapid development, with its most prominent advantage being the ability to achieve customized and small-batch production, independent of manufacturing costs and production volume. This is perfectly aligned with the need for small-batch and rapid customization of microfluidic chips, making the combination of 3D printing technology and microfluidic chip manufacturing a growing trend [13]. Furthermore, 3D printing technology, which has the capability to directly print three-dimensional models based on CAD technology, addresses the issue of pre-processing chip templates in traditional processes, which simplifies the manufacturing process and shortens the chip production cycle, enabling rapid response from design to manufacturing of microfluidic chips. Additionally, considering the small size of a fabricated microfluidic chip and the lower costs of printing materials, when compared from a manufacturing cost perspective, 3D printing technology also presents significant advantages in the low-cost production of microfluidic chips. Although various manufacturing methods for microfluidic chips based on different 3D printing principles have emerged, such as the production of droplet generators based on SLA (stereolithography apparatus) technology [14] and the fabrication of microfluidic capillary electrophoresis with integrated electrodes based on FDM (fused deposition modeling) technology [15], the equipment costs and printing time vary for different printing principles. It is necessary to select the appropriate 3D printing method in order to achieve the optimization of microfluidic chip cost and manufacturing efficiency while meeting the requirements for printing chip accuracy. The selection of a specific 3D printing solution for chip production needs to be considered comprehensively from various aspects, including manufacturing cost, printing accuracy, cost of printing materials, biocompatibility, and chemical resistance [16].

The extrusion-based printing method was widely used, primarily due to its versatility and low printing cost. And it has been extensively applied for fabrication of microfluidic chips in chemical and biomedical research [17,18,19]. The extrusion-based bioprinting method was utilized for the fabrication of organ-on-chips with sophisticated microstructures, which was promising in mimicking organ or tissues’ functions [20]. Organ-on-a-chip fabrication with simple one-step fabrication process was reported by Lee and Cho, which demonstrated the potential in producing organ-on-a-chips with 3D bioprinting [21]. FDM was also utilized to fabricate microfluidic chips because of its merits of low cost and ease of use, compared to other 3D printing technologies. For example, Duarte et al. designed microfluidic mixers containing 3D serpentine and Y-shaped microchannels, which were realized with FDM-based technology, and the device could be used for monitoring chemical reactions by mass spectrometry [22]. And transparent materials, including polystyrene, were used to improve the transparency of 3D-printed chips [23]. But the printing resolution of FDM-based technology limits its application in the fabrication of microfluidic chips with sub-100 μm microchannels, and it still needs efforts in improving the printing accuracy with FDM method.

Material jetting is another method that deposits tiny material drops to form microfluidic chips. Both PolyJet and MultiJet have successfully demonstrated the printing of high-precision microfluidic devices [24,25], but expensive printers, costly materials and post-treatment make it difficult for these technologies to be adopted by ordinary researchers. Compared to FDM technology, photopolymerization-based technology, including SLA and DLP (digital light processing), exhibits higher printing accuracy in chip printing and offers better printing quality for manufacturing three-dimensional microchannel structures. SLA is a type of photopolymerization 3D printing technology that utilizes a laser beam or UV beam to sequentially expose liquid photosensitive material, gradually solidifying it into the desired object. With the help of SLA technology, a 3D-printed microfluidic device with integrated valves and mixers was fabricated, and a culture chamber for analyzing signal propagation in HL-1 cardiomyocyte cell networks was also printed [26]. SLA technology offers higher print quality and detail accuracy, but it has a slower printing speed [27,28]. In particular, DLP technology adopts a mask exposure method, with each layer of curing time controlled within 10 s, enabling the completion of complex three-dimensional microfluidic chip printing within half an hour [29]. Thus, this technology could reach a balance between printing accuracy and printing time for microfluidic chip fabrication [30]. Zhang et al. utilized a DLP 3D printing method to make a microfluidic gradient concentration chip and achieved the accurate generation of on-chip antibiotic concentration gradients [31]. And by adopting a mask option strategy, high-accuracy, closed microchannels could be achieved with DLP 3D printers [32].

This study focused on the efficiency of chip printing, printing accuracy, and the manufacturing capability of complex chip structures. Based on DLP technology, this research investigated the process of direct printing integrated microfluidic chips. This study also demonstrated the manufacturing of complex microchannel structures by using transparent tape as a sealing material for open-channel 3D printing. Additionally, the reliability and cost-effectiveness of DLP technology in the 3D printing of microfluidic chips were specifically evaluated from the perspectives of printing accuracy, printing quality, material hydrophilicity/hydrophobicity, and material biocompatibility. Finally, by designing and printing a microfluidic shear stress chip, this study laid the foundation for the application of this process in areas such as cell culture and drug screening, showcasing the potential of DLP technology in printing microfluidic chips for biomedical applications.

## 2. Materials and Methods

### 2.1. Preparation of Microfluidic Chips with a DLP Printer

Starting from the goal of rapid customization of printed microfluidic chips, we have chosen a commercial DLP printer (MoonRay, SprintRay Co., Shaoxing, China) as the device for chip formation based on DLP printing technology. This printer uses a UV-LED that emits 405 nm wavelength ultraviolet light as the UV light source. Unlike common DLP light source devices on the market, the UV intensity of each pixel point projected onto the resin surface is higher, which facilitates the curing of photosensitive resin and reduces curing time. At an XY printing resolution of 75 μm, the printing size of the printer can reach 96 × 60 × 200 mm, and the minimum layer thickness in the Z direction is 20 μm, fully satisfying the requirements for printing conventional microfluidic chips.

**Figure 1:**
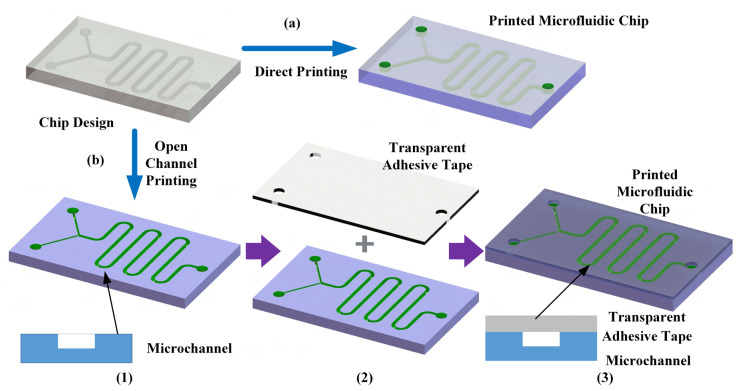
Chip manufacturing process based on DLP technology: (**a**) direct printing of integrated chips and (**b**) printing of open channel structures and chip formation achieved through transparent adhesive tape sealing.

According to the layer-by-layer printing characteristics of DLP photopolymerization technology, each layer of photosensitive resin is cured and formed under UV irradiation, eventually forming a three-dimensional structure. The curing of photosensitive resin and the elimination of residual resin are important steps in the photopolymerization process of microfluidic chip fabrication. The choice of different microfluidic chip structures and printing accuracies can affect the difficulty of printing microfluidic chips. For complex structures and printing of microchannels with diameters less than 200 μm, it is prone to channel blockage due to the difficulty of removing residual uncured photosensitive resin, resulting in chip manufacturing failure. Therefore, for different microfluidic chip designs, we proposed corresponding methods to manufacture microfluidic chips; namely, a direct printing method and open channel design with chip encapsulation using transparent adhesive tape, as shown in Figure 1. The process of a direct printing molding chip is as follows:

(1) Use three-dimensional modeling software, such as Solidworks (Solidworks 2021), to establish a three-dimensional model of the microfluidic chip. In the chip model, reserve structures such as chip interfaces. Import the chip model into the DLP printer software (Sprintray V2.15) in STL file format, and after layer slicing, obtain the printing control code.
(2) Depending on the application scenario of the printed chip, select suitable chip printing materials and machine printing accuracy settings. Use the obtained printing code to manufacture the chip.
(3) After the chip printing is completed, remove the chip from the printing platform and perform ultrasonic cleaning using a dedicated cleaning solution or anhydrous ethanol in an ultrasonic cleaner for 10 min.
(4) Rinse the residual cleaning solution or anhydrous ethanol from the chip’s surface with water and dry it by blowing or in a drying oven.
(5) Use 405 nm ultraviolet light for secondary curing of the chip and check the chip effect. Each irradiation lasts for 3 min. Repeat this process until the chip reaches the best state. Save the microfluidic chip for future use.

For most chip designs, direct printing is a feasible method for chip fabrication. Its major advantages include simple and practical steps, short average printing time, and ease of use for microfluidic researchers to quickly grasp the chip printing process and develop corresponding applications. However, direct printing of chips may encounter difficulties in printing certain chips due to extremely small microchannel diameters or complex channel structures. Considering the requirement of chip material transparency, an alternative method to complement direct chip printing is to use an open channel design with transparent adhesive tape encapsulation. The specific process is as follows (shown in Figure 1b):

(1) Based on the initial design of the microfluidic chip, modify the channel design from resin encapsulation to an open channel design, and reserve a flat structure for channel encapsulation.
(2) Import the open channel model into the DLP printing software and generate the printing code.
(3) Depending on the application scenario of the printed chip, select suitable chip printing materials and printing accuracy settings. Especially for microstructures, choose the appropriate printing direction to avoid structural distortion caused by improper selection of the Z printing direction. Use the obtained printing code to manufacture the chip.
(4) After the chip printing is completed, remove the chip from the printing platform and perform ultrasonic cleaning using a dedicated cleaning solution or anhydrous ethanol in an ultrasonic cleaner for 10 min. Then rinse the residual cleaning solution or anhydrous ethanol from the chip’s surface with water and blow dry, or dry in a drying oven.
(5) Use a 405 nm ultraviolet light for secondary curing of the chip and check the chip effect. Each irradiation lasts for 3 min. Repeat this process until the chip reaches the optimal state.
(6) Place the chip after secondary curing in close contact with transparent adhesive tape (Scotch, 3M, Tonawanda, NY, USA) and remove any air bubbles between the bonding surfaces to ensure a flat and clean encapsulation plane.
(7) Heat the encapsulated chip in an oven at a temperature lower than the resin’s thermal deformation threshold for 1–2 h to ensure stable bonding. After confirming the encapsulation effect, retain the chip.

The overall manufacturing process using the open channel design with transparent adhesive encapsulation is similar to the direct printing method, but with improved chip design and an additional encapsulation step. The advantage of this approach is its ease of removing residual resin and the ability to fabricate high-precision microchannel structures. Furthermore, the use of transparent adhesive facilitates optical observation and detection of the microfluidic chip, addressing the issue of poor transparency in resin chips.

### 2.2. Characterization of Printing Accuracy of Microfluidic Chips

**Figure 2:**
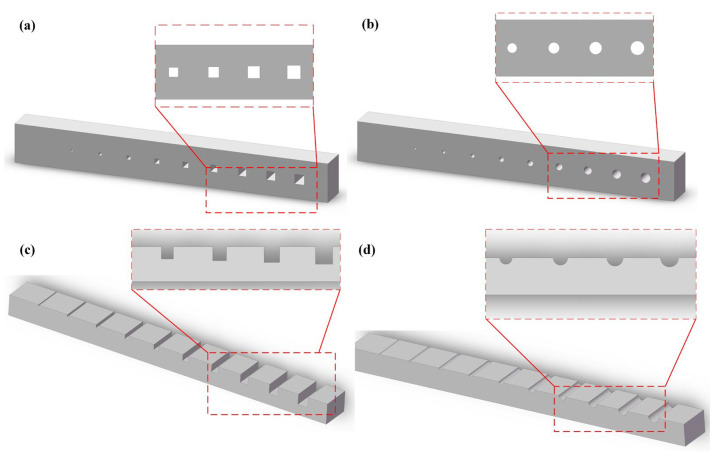
Characterization of the microfluidic chip’s printing accuracy: (**a**,**b**) schematic diagrams of square and circular microchannel designs based on DLP technology for direct printing and (**c**,**d**) schematic diagrams of square and semi-circular open microchannels based on an open channel design.

The structure of microfluidic chips varies depending on different application requirements. The ability to manufacture chips in a 1:1 ratio according to the design determines the overall performance of the microfluidic chip. In DLP printing of microfluidic chips, the accuracy of the overall chip formation is determined by the printer’s accuracy and specific printing settings. In our study, we specifically investigated and analyzed the manufacturing accuracy of microchannels under two manufacturing processes of DLP-printed microfluidic chips: direct printing and printing with an open channel design followed by encapsulation. In the direct printing process of microfluidic chips, we used the printed channel cross-sections to verify the printing accuracy. We selected two typical channel cross-sections, namely square and circular, and measured and statistically analyzed the formed channel dimensions. The chip design representation is shown in Figure 2a,b. We divided the plane where the channel cross-section was located into XY directions and measured the channel dimensions in the X and Y directions, corresponding to the width and height of the microchannel, respectively. By comparing the measured dimensions with the designed dimensions of the microchannel and calculating the corresponding relative error values, we analyzed the printing accuracy in detail. For the design of open channels, it is necessary to consider the actual achievable channel cross-sections. In this study, we selected square and semicircular channel cross-sections for specific analysis and printed the corresponding chips. The chip design representation is shown in Figure 2c,d. And 3D-printed samples for the test are shown in Figure S1. The DLP printer’s official printing software is not open source, and the printing mode could only be set as high-, medium-, or low-quality. To exclude the effect of printing settings, we chose the high-quality mode in the printing software. The layer thickness in the Z direction was set as 20 μm to ensure the best printing performance.

### 2.3. Characterization of Surface Quality of 3D-Printed Chips

According to the characteristics of the DLP printing process, each layer of the solidified structure is formed by projecting the projection surface onto the forming interface in a single step, and the formed layers are stacked incrementally along the Z direction to obtain the solid model. Therefore, the printing direction in the Z direction determines the direction of the step pattern on the formed chip, i.e., the step pattern direction inside the microchannel varies with different printing directions, resulting in different surface qualities of the microchannel. Under the condition where the microchannel direction was parallel to the printing Z-direction, the Z-layer thickness was set as 20 μm, 50 μm, and 100 μm. And under the condition where the microchannel direction was perpendicular to the printing Z-direction, the Z-layer thickness was set as 20 μm. The surface quality of the printed microfluidic chip was measured using an optical microscope and a coherence scanning interferometer (NewView 8200, Zygo Corporation, Middlefield, CT, USA).

### 2.4. Characterization of Printing Materials for 3D-Printed Chips

The hydrophilicity or hydrophobicity of chip materials is also a factor to consider in microfluidic chip design. The hydrophilicity or hydrophobicity of a material is quantitatively analyzed based on the contact angle. When the contact angle *θ* ≤ 90°, it is defined as essentially hydrophilic, while when *θ* > 90°, it is defined as essentially hydrophobic. For microfluidic chips printed in different directions, we utilized a contact angle measurement device (DropMeter A-100P, MAIST Vision Inspection & Measurement Co., Ltd., Ningbo, China) to precisely measure their contact angles and analyze the hydrophilicity or hydrophobicity of the printing materials.

In basic applications such as cell culture in microfluidic chips, cells are highly sensitive to the toxicity of materials, and good biocompatibility of materials is beneficial for cell proliferation. We used the SP-RD202 (SprintRay Co., Shaoxing, China) photosensitive resin as the printing material for the microfluidic chips and produced multiple sets of identical microfluidic chips as the subject of the study on the biocompatibility of the resin material. By conducting cell culture using the microfluidic chips and analyzing cell viability, we investigated the biocompatibility of this resin.

Firstly, HUVEC (human umbilical vein endothelial cells) were seeded in an untreated 96-well plate and in a 96-well plate treated with GelMA (gelatin methacrylate) gel as the control group. GelMA, known for its excellent biocompatibility, is conducive to cell adhesion and proliferation. In this experiment, GelMA was used as the surface treatment material on the chip to enhance the cultivation performance. The dimensions of the microfluidic chip culture chambers were the same as those of each well in the 96-well plate. The printed resin chips were either left untreated or surface treated with a coating of GelMA gel. HUVEC with the same density were then seeded on these two types of treated microfluidic chips as the experimental groups. The quantity of viable cells was determined using a CCK-8 assay kit (Solarbio, Beijing, China), measuring the optical density (OD) at 450 nm using a microplate reader. The cell viability values of the corresponding experimental groups were calculated using the formula: Cell viability (%) = [A(experimental group) − A(blank)]/[A(control group) − A(blank)] × 100.

## 3. Results and Discussions

### 3.1. Analysis of Printing Accuracy of Microfluidic Chips

We conducted a detailed study to analyze the manufacturing accuracy of microchannels using two different methods: direct printing and printing followed by encapsulation of open channels. We divided the plane where the channel cross-section was located into the XY directions and measured the dimensions of the channel in both X and Y directions, corresponding to the width and height of the microchannel, respectively. By comparing the designed dimensions of the microchannels with the actual measured dimensions and calculating the corresponding relative error values, we were able to analyze the printing accuracy.

**Figure 3:**
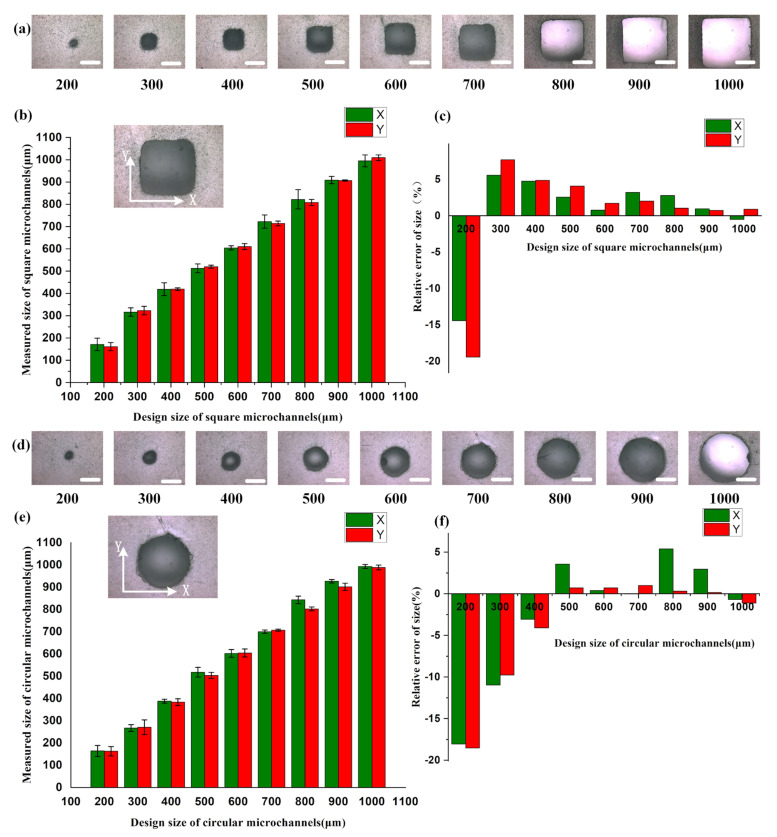
Characterization of printing accuracy of directly printed square and circular microchannels: (**a**) cross-section of the square microchannels with a scale bar of 300 μm; (**b**) comparison between the chip design dimensions and the actual dimensions, with the measured data representing the dimensions of the channel cross-section in the X and Y directions (error bars indicate the arithmetic mean ± standard deviation of each data set); (**c**) relative error values of the printed square microchannels; (**d**) cross-section of the circular microchannel with a scale bar of 300 μm; (**e**) comparison between the chip design dimensions and the actual dimensions, with the measured data representing the dimensions of the channel cross-section in the X and Y directions (error bars indicate the arithmetic mean ± standard deviation of each data set); and (**f**) relative error values of the printed circular microchannels.

Figure 3a shows the square microchannels printed using the direct printing method. The minimum achievable size is 200 μm, which is dependent on the accuracy and resolution of the DLP printer. Improving the pixel resolution of the DMD (digital micromirror device) can enhance manufacturing accuracy. When the channel design size is 300 μm or larger, the channel cross-sections are more regular and square-shaped. As the channel design size increases, the overall channel size error gradually decreases, indicating an improvement in printing accuracy. The overall aspect ratio of the cross-sections X:Y tends to approach 1:1. The relative size error in the X direction is controlled within 10% when the design size is greater than 300 μm, as is the case for the Y direction. At the minimum achievable size of 200 μm, the manufacturing error can still be controlled within 20%. Similarly, for directly printed circular microchannels, the minimum achievable size is 200 μm (Figure 3d). The overall aspect ratio of the cross-sections X:Y also tends to approach 1:1, indicating a more complete circular cross-section. However, considering the impact of residual resin, the size error in both the X and Y directions is approximately 20% for a design size of 200 μm and around 10% for a design size of 300 μm. When the design size exceeds 400 μm, both the X and Y direction size errors can be controlled within 5%. Therefore, the direct printing method demonstrates higher printing accuracy for microchannels with a size equal to or larger than 300 μm.

**Figure 4:**
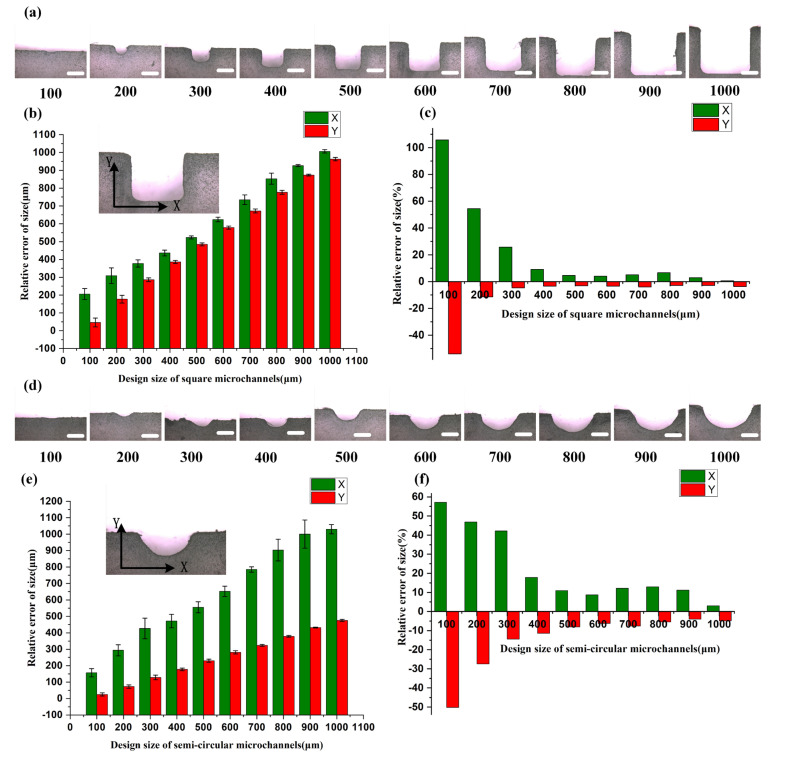
Characterization of printing accuracy of square and semi-circular microchannels with open channel design: (**a**) cross-section of the square microchannels with a scale bar of 300 μm; (**b**) comparison between the chip design dimensions and the actual dimensions, with the measured data representing the dimensions of the channel cross-section in the X and Y directions (error bars indicate the arithmetic mean ± standard deviation of each data set); (**c**) relative error values of the printed square microchannels; (**d**) cross-section of the semi-circular microchannel with a scale bar of 300 μm; (**e**) comparison between the chip design dimensions and the actual dimensions, with the measured data representing the dimensions of the channel cross-section in the X and Y directions (error bars indicate the arithmetic mean ± standard deviation of each data set); and (**f**) relative error values of the printed semi-circular microchannels.

As shown in Figure 4, both the square and semi-circular microchannels exhibit regular cross-sections, with the overall aspect ratios in the width and height directions, X:Y, approaching 1:1 and 2:1, respectively. Regardless of the design of square or semi-circular microchannels, the size error in the X direction is positive, indicating that the actual manufactured size in the X direction is larger than the design size. Conversely, the size error in the Y direction is negative, indicating that the actual size in the Y direction is smaller than the design size. In the case of square microchannel designs, although the minimum channel size can reach 100 μm, there is significant manufacturing error within the range of 300 μm and below. Only when the design size is larger than 400 μm, can both X and Y direction errors be controlled within 10%. Particularly, within the range of 100–200 μm, there is considerable size error in the X direction, which may be due to limitations in the pixel resolution of the projection, making it difficult to accurately solidify the resin at the edges of the open channels. Regarding the semi-circular microchannel designs, the difficulty in accurately shaping the channel edges leads to large overall size errors, exceeding 20%, when the design size is smaller than 400 μm. Only when the design size is at 500 μm or above, can the size errors be controlled within approximately 10%.

Therefore, for open microchannel designs, the accuracy of the channel formed at the interface with the transparent sealant is less than that of directly printed channels, due to the limitations of the resolution of the DLP printer. In practical design, it is necessary to make the appropriate advance error compensation for channel sizes based on the chip manufacturing method. For a microchannel with a size of over 300 μm via a direct printing option and a microchannel with a size of over 500 μm via an open channel design, the printer could achieve good printing accuracy. When the microchannel size is lower than 300 μm for direct printing and 500 μm for open channel design, it is recommended that extra error compensation is utilized before the chip’s final design. And the percentage of the microchannel size compensation could be made based on the experimental results shown in Figure 3 and Figure 4. Overall, the printing accuracy of chip fabrication based on the DLP process can meet the requirements of most applications.

### 3.2. Analysis of Surface Quality of Printed Chips

**Figure 5:**
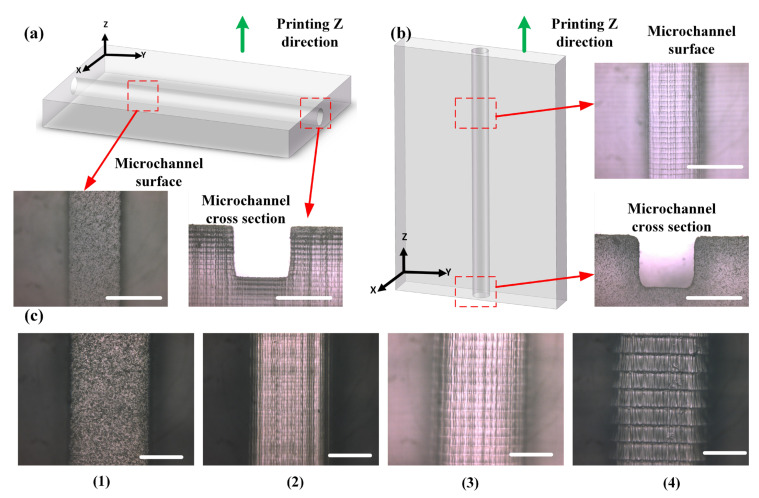
Influence of chip printing direction and layer thickness in the printing Z direction on the surface quality of the chip: comparison of chip surface quality under two conditions, including a microchannel direction perpendicular to the Z direction of printing (**a**) and a microchannel direction parallel to the Z direction of printing (**b**), scale bar = 500 μm; (**c**) Microchannel morphology with microchannel direction perpendicular to the Z direction of printing (**1**) and microchannel direction parallel to the Z direction of printing with layer thicknesses of 20 μm (**2**), 50 μm (**3**), and 100 μm (**4**), scale bar = 200 μm.

As shown in Figure 5a,b, based on the relationship between the direction of fluid flow (i.e., the microchannel direction) and the printing Z-direction, there are two main relationships: perpendicular and parallel. The formation in the inclined direction lies between these two relationships. Under the condition where the microchannel direction is parallel to the printing Z-direction, it can be further divided into three categories based on the specific value of the Z-layer thickness in the parameters of the DLP printer, namely 20 μm, 50 μm, and 100 μm. The Z-layer thickness represents the height by which the printing platform is raised after each layer solidification, and this is also related to the spacing size of the step pattern.

Figure 5c illustrates the surface morphology of the microchannel when the microchannel direction is perpendicular to the printing Z-direction. Due to the single-step formation of the overall surface, it appears relatively smooth. On the other hand, Figure 5c shows the microchannel surfaces at different Z-layer thicknesses when the microchannel direction is parallel to the printing Z-direction. Since the Z-layering is denser, the surface quality of microchannels with a Z-layer thickness of 20 μm is better than that with a Z-layer thickness of 100 μm. Based on the measurement results of a coherence scanning interferometer, as shown in Figure S2, it can be observed that when the microchannel direction is perpendicular to the printing Z-direction, the surface roughness is minimal, with a surface roughness (Sa) of 1.303 μm. Meanwhile, when the microchannel direction is parallel to the printing Z-direction, the surface roughness is relatively higher. Specifically, the surface quality is similar for Z-layer thicknesses of 20 μm and 50 μm, with Sa values of 2.739 μm and 2.394 μm, respectively. At a Z-layer thickness of 100 μm, the surface is roughest, with an Sa value of 3.550 μm. Therefore, considering the characteristics of the printing direction, a larger spacing between step patterns results in a rougher surface. When manufacturing microfluidic chips, it is advisable to prioritize adjusting the printing direction of the chips and use a smaller Z-layer thickness to achieve higher surface quality.

### 3.3. Analysis of Printing Materials’ Properties

**Figure 6:**
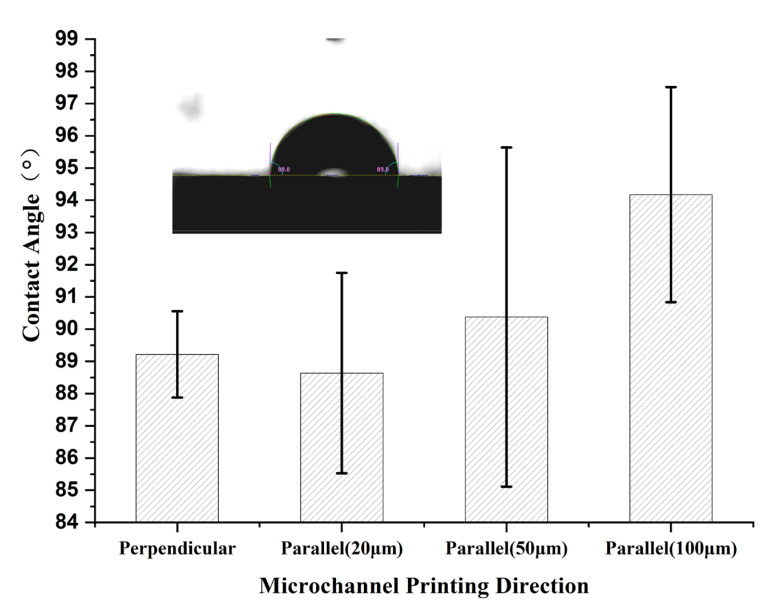
The contact angles of the chip materials were measured under different printing conditions. Included in this figure, from left to right, are the microchannel direction perpendicular to the printing Z direction, the microchannel direction parallel to the printing Z direction with printing layer thicknesses of 20 μm, 50 μm, and 100 μm, respectively (error bars represent the arithmetic mean ± standard deviation of each data set).

For the microfluidic chips under different printing directions, we specifically measured their contact angles and analyzed the hydrophilicity/hydrophobicity of the materials, as shown in Figure 6. In both cases where the microchannel direction is perpendicular to the printing Z-direction and parallel to the printing Z-direction (with a layer thickness of 20 μm), the contact angles are less than 90°, indicating hydrophilicity. However, in the other two cases, where the microchannel direction is parallel to the printing Z-direction (with layer thicknesses of 50 μm and 100 μm), the contact angles are greater than 90°, indicating hydrophobicity. The same material exhibits different contact angles and shows both hydrophilic and hydrophobic behavior. This phenomenon can be attributed to the analysis of the real surface, where the physical or chemical defects result in a complex surface condition, especially on rough surfaces, and the wetting characteristics are related to surface roughness. According to the Wenzel equation [33], in this state, it can be deduced that, on a rough surface, the contact angle will decrease as the roughness increases when the smooth surface contact angle is less than 90°, while on a smooth surface, the contact angle will increase with increasing roughness when the smooth surface contact angle is greater than 90°. Considering the previously summarized surface roughness under different printing directions, it is in line with this theory that the roughness perpendicular to the Z-direction is smaller than that parallel to the Z-direction (20 μm), and the roughness of the parallel Z-direction (20 μm) slightly decreases. On the other hand, the roughness of the parallel Z-direction (100 μm) is greater than that of the parallel Z-direction (50 μm), and the corresponding contact angle also increases. However, despite these observations, the experimentally measured contact angles of the materials are close to 90°. In practical applications, the surface can be modified to achieve the desired hydrophilic or hydrophobic state according to the requirements of surface wetting properties.

**Figure 7:**
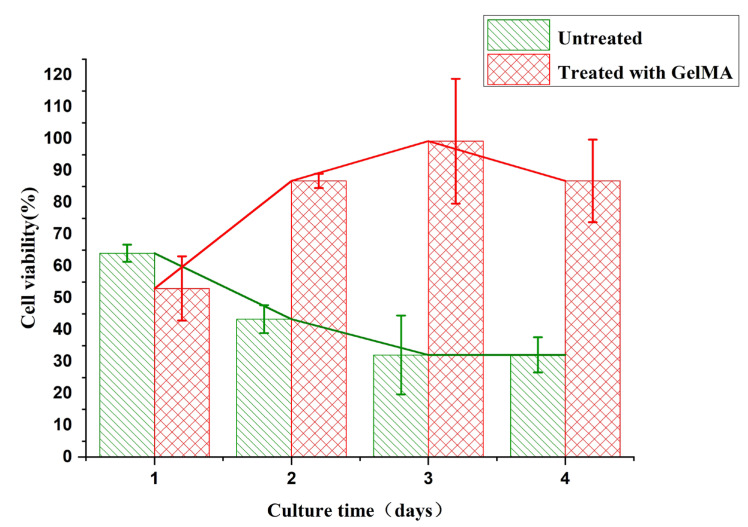
Analysis of biocompatibility of chip materials: cell viability analysis on the surface of untreated chips and GelMA treated chips (error bars represent the arithmetic mean ± standard deviation of each data set).

Figure 7 shows the changes in cell viability within 1 to 4 days on the surfaces of untreated resin material and GelMA treated gel surfaces. From the experimental results, it can be observed that the resin material chip without any treatment exhibits certain toxicity to the cells. The cell viability decreases from 64.03 ± 2.70% on the first day to 32.13 ± 5.56% on the fourth day. In contrast, the chip treated with GelMA gel shows an increase in cell viability from 52.98 ± 10.08% on the first day. On the 2nd, 3rd, and 4th days, the cell viability remains above 85%, indicating that GelMA gel treatment can reduce the cytotoxic effect of the chip material on the cells. However, on the first day, the cell viability of the GelMA-treated group is slightly lower than that of the untreated group. This may be due to the fact that HUVEC cells require some time for proliferation and spreading on the GelMA gel surface, which is slower compared to the surface of the chip [34,35,36]. Therefore, the SP-RD202 resin material exhibits some toxicity, which may be related to incomplete photopolymerization of the resin or the presence of toxic materials in the resin formulation. However, the cell culture performance of the resin material can be improved by employing resin surface treatment methods.

### 3.4. Three-Dimentional-Printed Microfluidic Chips and Their Applications

**Figure 8:**
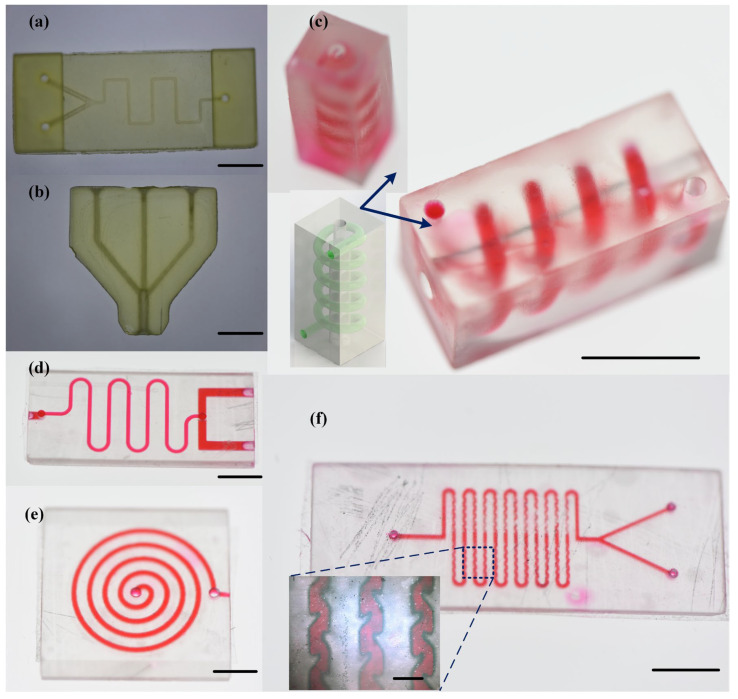
Demonstration of 3D-printed 2D and 3D microfluidic chips with a DLP-based printer: (**a**,**b**) directly printed 2D microfluidic chips, scale bar = 5 mm; (**c**) directly printed 3D microfluidic chip, with a three-dimensional helical channel structure, scale bar = 10 mm; (**d**,**e**) microfluidic chips based on an open-channel design and transparent encapsulation with adhesive tape, scale bar = 5 mm; and (**f**) micro-mixer based on an open-channel design and transparent encapsulation with adhesive tape, scale bar = 5 mm, with the locally magnified image showing the details of the channel structure, scale bar = 400 μm.

Following the printing process described earlier, two-dimensional (2D) and three-dimensional (3D) microfluidic chips with different channel structures were manufactured using both direct printing and open-channel printing methods. Figure 8a,b shows 2D microfluidic chips directly manufactured using a DLP printer, demonstrating that most conventional chip designs can be directly manufactured using the DLP printing process. The greatest advantage of 3D printing technology lies in its ability to construct three-dimensional microchannel structures. Figure 8c shows a microfluidic chip with a three-dimensional spiral channel structure, which was achieved through the DLP process to create three-dimensional curved surfaces, a task that was difficult to achieve with traditional fabrication methods. Three-dimensional microfluidic chips outperform two-dimensional microfluidic chips in terms of detection capability and sample handling. The methods described in this paper provide a more convenient way to design and validate the functional structures of 3D microfluidic chips, and the achieved 3D structures differ from those created using traditional stacking methods.

In direct printing of microfluidic chips, residual resin removal has always been a key issue affecting printing accuracy and channel integrity. However, the open channel design proposed in this paper effectively addresses these issues. Open channels are more easily flushed with anhydrous ethanol or cleaning solutions, effectively solving the problem of resin residues blocking the channels, especially when the channel size is too small for effective cleaning. Figure 8d,e demonstrates two different microfluidic chips based on an open-channel design and transparent encapsulation. The former had conventional closed-channel designs at the channel interfaces for easy connection to tubing, while the middle section of the channels was open, allowing for the printing of smaller-sized microchannels. Both these chips maintained a sealed and stable condition under normal pressure, without any leakage issues. Figure 8f illustrates the manufacturing advantages of combining the open channel design with transparent encapsulation on complex and intricate microstructures. By introducing intricate and winding microchannel configurations based on conventional micro mixers, the fluid mixing effect is enhanced, effectively improving the mixing performance of the micro mixer.

To meet the design requirements of different types of microfluidic chips, the design and manufacturing of microfluidic chips were achieved using the DLP printing process. Fluid shear stress is an important indicator of blood flow in human blood vessels. By simulating the effect of different levels of fluid shear stress on corresponding cells using microfluidic chips, particularly HUVEC (human umbilical vein endothelial cells), cellular morphology changes can be observed. Additionally, different magnitudes of fluid shear stress can affect the expression of genes in endothelial cells. Therefore, combining numerical simulation software (ANSYS), we proposed a microfluidic chip design for fluid shear stress (shown in Figure S3). By using ANSYS Workbench, the simulation analysis could be finished efficiently, and details about the numerical model are presented in the Supporting Information (Figures S3 and S4).

**Figure 9:**
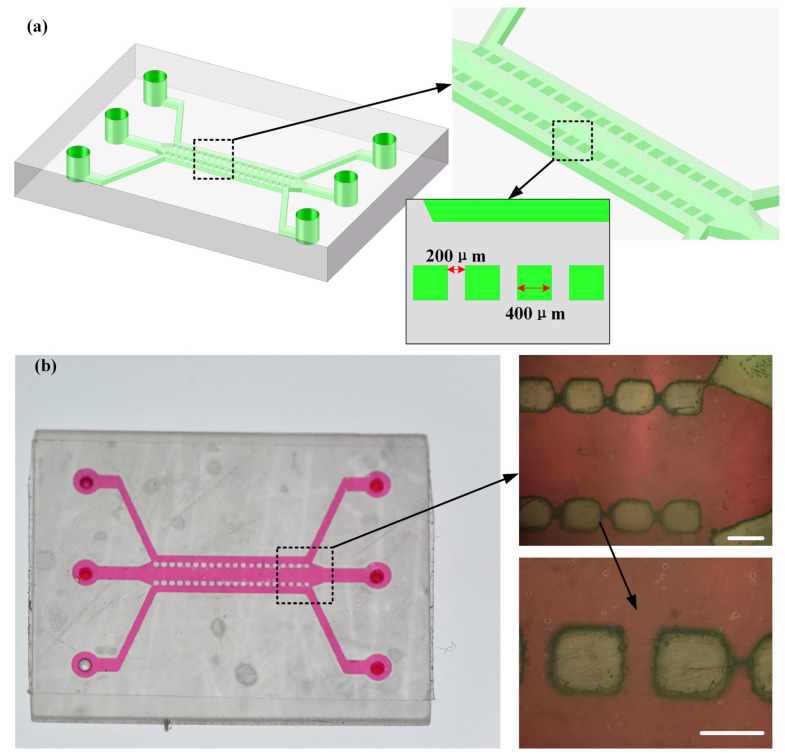
Design and fabrication of microfluidic shear stress chip: (**a**) design of microfluidic shear stress chip and (**b**) image of the fabricated microfluidic chip, scale bar = 400 μm in both locally magnified images.

Figure S4a illustrates the initial design model of the microfluidic chip. The central channel was used for applying fluid shear stress, while the two side channels served as cell seeding and dynamic perfusion channels. Different cell types were seeded in the side channels to establish a co-culture model, allowing the study of the effects of cellular products on the directed differentiation of endothelial cells. Based on the initial model, we set different perfusion flow rates to investigate the distribution of wall shear stress inside the chip. Figure S4b–f shows the wall shear stress distribution for flow rates of 10 mL/h, 25 mL/h, 50 mL/h, 75 mL/h, and 100 mL/h, respectively. Combining the results from numerical simulations and optimizing the chip dimensions, we obtained the microfluidic chip design shown in Figure 9. By employing the open channel design proposed in this paper, complex and intricate microstructures, as demonstrated in Figure 9b, can be fabricated on the chip surface. The chip was then manufactured by transparent encapsulation with adhesive tape. This design of the microfluidic chip serves as a research platform for studying fluid shear stress and has potential applications in the simulation of in vitro vascular models and endothelial cell differentiation studies.

**Figure 10:**
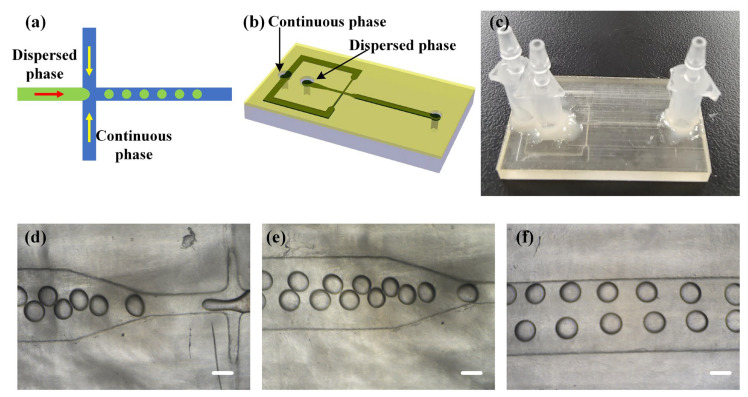
The 3D-printed microfluidic chip for hydrogel microsphere generation: (**a**) principle of hydrogel microsphere generation; (**b**) design of microfluidic chip for hydrogel microsphere generation; (**c**) 3D-printed microfluidic chip with transparent adhesive tape encapsulation; (**d**) image of the generation process of hydrogel microspheres, scale bar = 200 μm; (**e**) image of generated hydrogel microspheres, scale bar = 200 μm; and (**f**) image of hydrogel microspheres in the collecting zone, scale bar = 200 μm.

Hydrogel microspheres are widely used as novel functional materials in various fields such as biology and medicine. Microfluidics, due to its advantages in fluid manipulation, is used to generate uniform microspheres with controlled morphology [37]. In traditional PDMS (polydimethylsiloxane) chips, the transparent property of PDMS allows for easy observation of the microsphere formation process and measurement of the microspheres. However, in traditional 3D-printed microfluidic chips, the chip materials often have limited transparency, which hampers the observation effect. In this study, an open channel design with a transparent adhesive tape encapsulation method is proposed, where a transparent adhesive tape is used as the encapsulation material, enabling good light transmission. It was employed to design and print a hydrogel microsphere generation chip, as shown in Figure 10. Mineral oil (Kehbio, Beijing, China) was used as the continuous phase at a flow rate of 8.8 μL/min, while poly(ethylene glycol) diacrylate (PEGDA) with an average molecular weight of ~200 (Aladdin, Wuhan, China) was used as the dispersed phase at a flow rate of 2.2 μL/min. This setup allowed for the stable generation of hydrogel microspheres with a diameter of 300 μm (shown in Figure 10d,f and Video S1).

## 4. Conclusions

In this study, we investigated an integrated printing process for resin-based microfluidic chips based on 3D printing equipment utilizing DLP technology. This process demonstrates significant advantages in terms of manufacturing efficiency and cost-effectiveness for microfluidic chip production. The research findings regarding the chip printing process and chip performance are as follows: (1) DLP printing technology can meet the precision requirements for most microfluidic chips. However, error compensation methods need to be introduced for printing microchannels with sizes below 300 μm to ensure the printing accuracy of microfluidic chips. (2) Selecting appropriate printing direction and parameter of Z-layer thickness for the chip can improve the surface quality and wettability. (3) The biocompatibility of resin chip materials is somewhat limited, but it can be improved through surface treatment methods. Based on the above process research, we successfully designed and manufactured a microfluidic chip for fluid shear stress, demonstrating the potential applications of 3D-printed chips in the biomedical field.

## Data Availability

Data available upon request.

## Supplementary Materials

The following supporting information can be downloaded at: https://www.mdpi.com/article/10.3390/ma16216984/s1, Figure S1: Characterization of microfluidic chip’s printing accuracy; Figure S2: Surface quality of the chip with different printing directions and layer thickness; Figure S3: Design of microfluidic chip for fluid shear stress; Figure S4: Design of microfluidic shear stress chip; Video S1: Formation of hydrogel microspheres with 3D-printed microfluidic chip.
