# Supplementary material for: 3D Printing of Individualized Microfluidic Chips with DLP-Based Printer
Source: Materials (Basel). 2023 Oct 31;16(21):6984. doi: 10.3390/ma16216984 (PMC10650121; doi:10.3390/ma16216984)
Supplement: Supplementary file 1 [file materials-16-06984-s001.zip › Supplementary File-Materials-R2.pdf]

Supporting Information

3D printing of individualized microfluidic chips with DLP-based printer

Jingjiang Qiu [1,2,3,*], Junfu Li [1], Zhongwei Guo [1,2,3], Yudong Zhang [1,2,3], Bangbang Nie [1,2,3], Guochen Qi [1,2,3], Xiang Zhang [1], Jiong Zhang [4] and Ronghan Wei [1,2,3,5,*]

[1] School of Mechanics and Safety Engineering, Zhengzhou University, Zhengzhou 450001, China;
[2] Engineering Technology Research Center of Henan Province for MEMS Manufacturing and Applications, Zhengzhou University, Zhengzhou 450001, China;
[3] Institute of Intelligent Sensing, Zhengzhou University，Zhengzhou 450001, China;
[4] Department of Mechanical Engineering, College of Engineering, City University of Hong Kong, Kowloon Tong, Kowloon, Hong Kong, China;
[5] Industrial Technology Research Institute, Zhengzhou University, Zhengzhou 450001, China;
* Correspondence: qjjzzu@zzu.edu.cn(J.Q.); profwei@zzu.edu.cn(R.W.)

S1. Characterization of printing accuracy of microfluidic chips

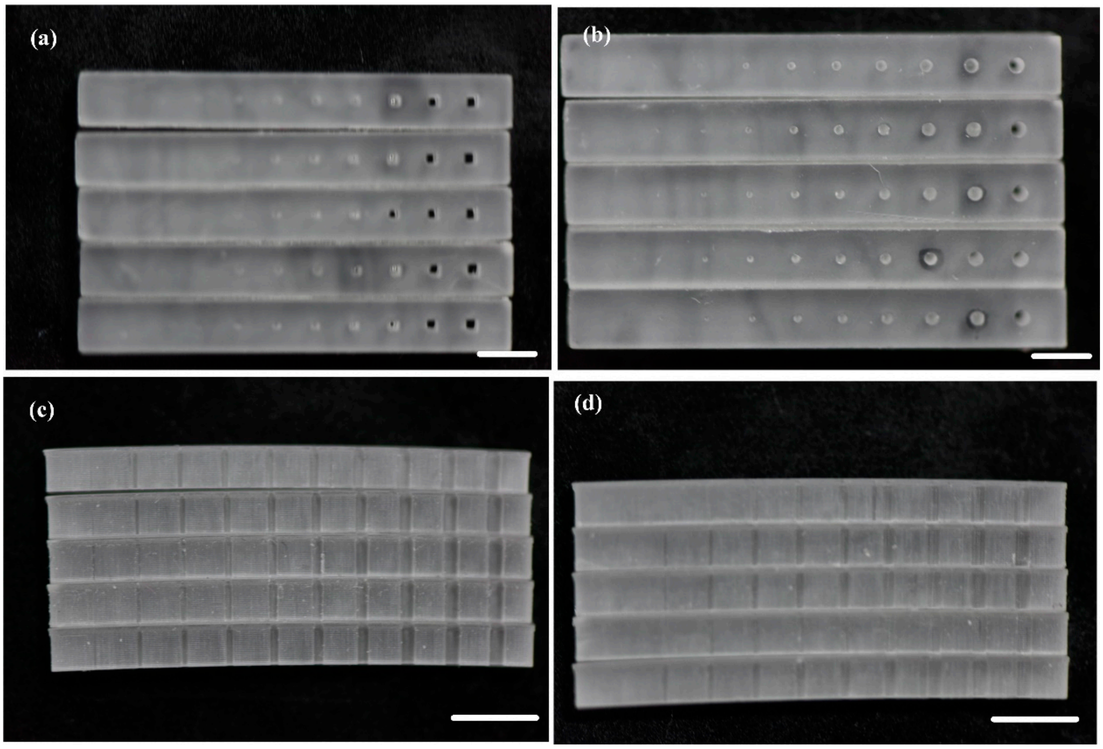

Figure S1. Characterization of microfluidic chip's printing accuracy: (**a**) & (**b**) Images of printed chips of square and circular microchannel designs based on DLP technology for direct printing, scale bar=5 mm; (**c**) & (**d**) Images of printed chips of square and semi-circular open microchannel designs based on open channel design, scale bar=10 mm.

S2. Analysis of surface quality of 3D-printed chips

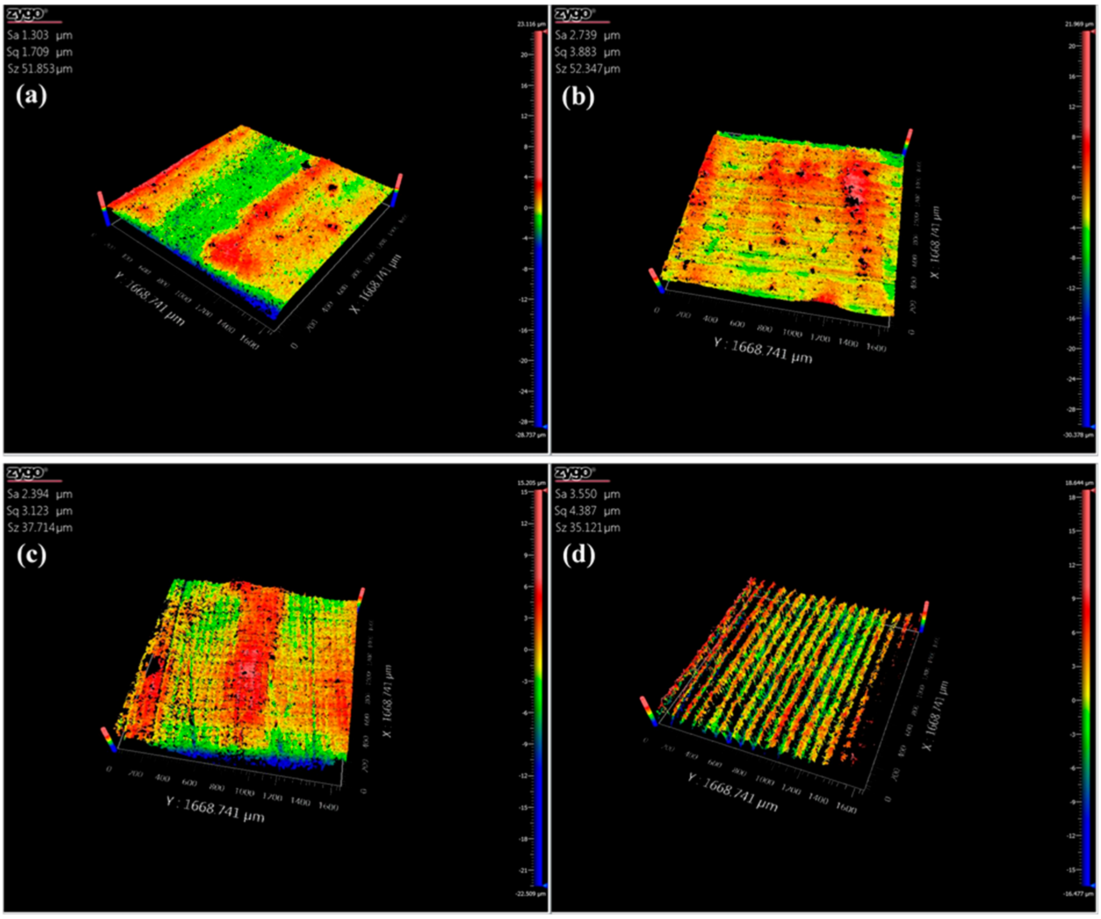

Figure S2. Surface quality of the chip with different printing directions and layer thickness: Under coherence scanning interferometer measurement, surface of the microchannel with microchannel direction perpendicular to the Z direction (**a**), and surface of the microchannel with microchannel direction parallel to the Z direction and layer thicknesses of 20 µm (**b**), 50 µm (**c**), and 100 µm (**d**).

S3. Microfluidic chip design for fluid shear stress research

Combining numerical simulation software (ANSYS), we proposed a microfluidic chip design for fluid shear stress, and all the simulation work was finished via ANSYS Workbench. The design of microfluidic chip could be found in Figure S3a, and inlet 2 was set for fluid shear stress loading while inlet 1&3 were set for culture medium perfusion. And the microfluidic chip consisted of 3 parallel channels separated by micropillar arrays. The detailed sizes for microchannels and micropillar arrays were presented in Figure S3b. The micropillar was square with a size of 400 µm. After the numerical model was transferred into ANSYS Workbench, the meshing work was finished first before setting the boundary parameters. The meshing parameters could be automatically provided by the software, but we rechecked the girds and revised several parameters to get more accurate results (Figure S3c). Sizing setting was changed to On: Proximity and Curvature, and Relevance Center was changed to Fine. Smoothing setting was also chosen as high. Min Size was kept as 4.2115e-006m while Max Size was kept as 8.423e-004m. After meshing, the simulation was done under no-slip boundary condition. By using ANSYS Workbench, the simulation analysis could be finished efficiently and results were presented in the Figure S4.

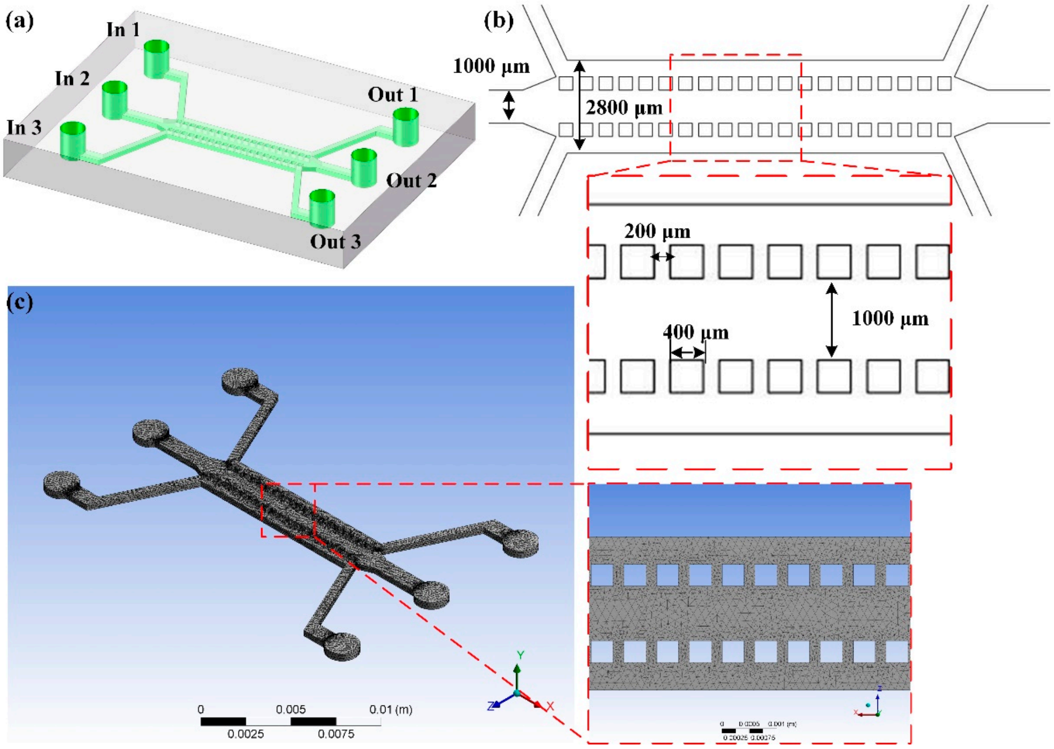

Figure S3. Design of microfluidic chip for fluid shear stress: (**a**) Design of microfluidic chip model, including inlet 2 for fluid shear stress loading and inlet 1&3 for culture medium perfusion; (**b**) Detailed size of microstructures in the microfluidic chip; (**c**) Demonstration of grids after the model was meshed in ANSYS Workbench.

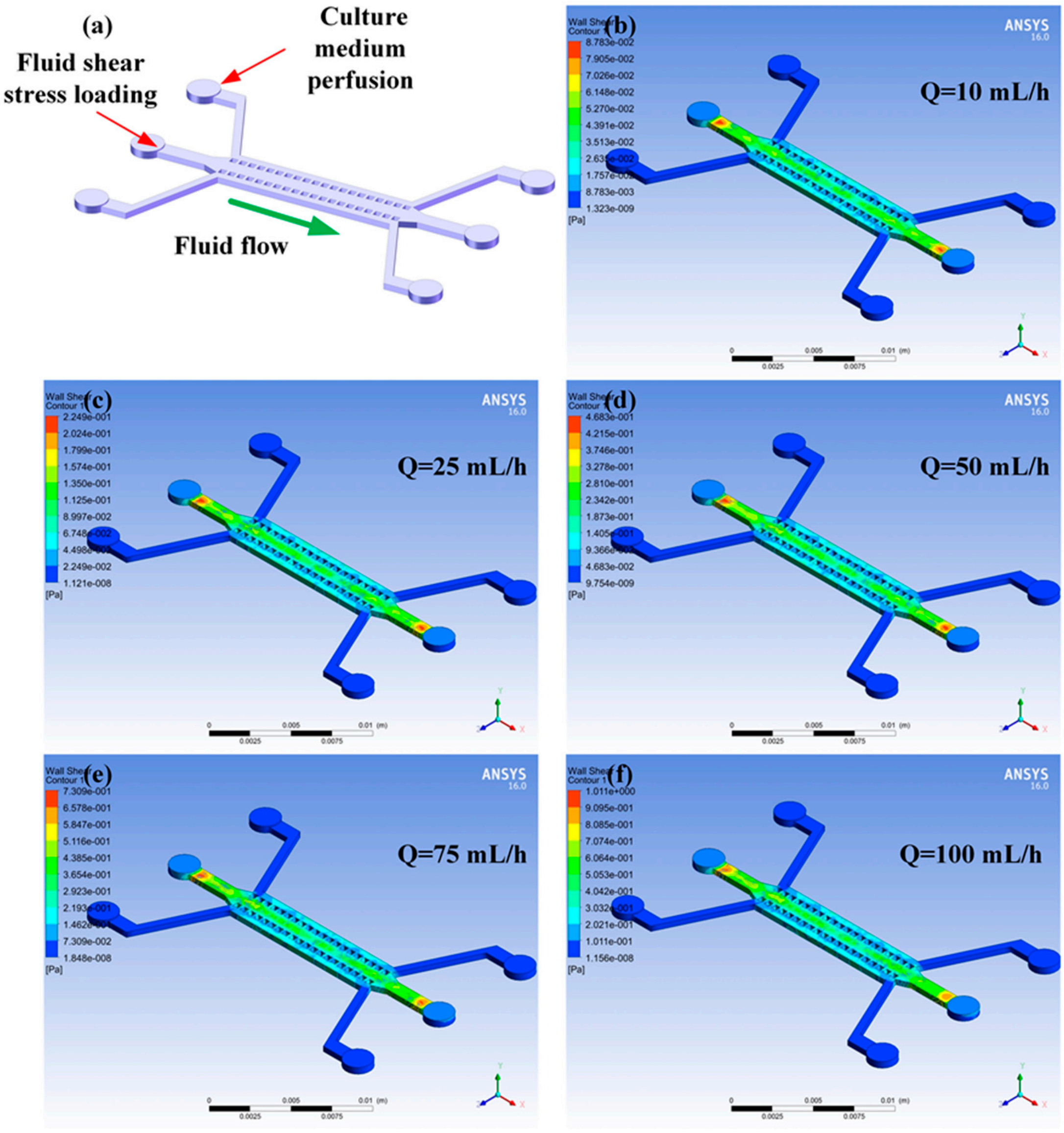

Figure S4. Design of microfluidic shear stress chip: (**a**) Microfluidic chip model; (**b**)-(**f**) Wall shear stress distribution simulation results under flow rates of 10 mL/h, 25 mL/h, 50 mL/h, 75 mL/h, and 100 mL/h.
